# Gene Expression Profiling Identifies Microphthalmia-Associated Transcription Factor (MITF) and Dickkopf-1 (DKK1) as Regulators of Microenvironment-Driven Alterations in Melanoma Phenotype

**DOI:** 10.1371/journal.pone.0095157

**Published:** 2014-04-14

**Authors:** Mariusz L. Hartman, Beata Talar, Muhammad Zaeem Noman, Anna Gajos-Michniewicz, Salem Chouaib, Malgorzata Czyz

**Affiliations:** 1 Department of Molecular Biology of Cancer, Medical University of Lodz, Lodz, Poland; 2 Unité INSERM U753, Institut de Cancérologie Gustave Roussy, Villejuif, France; Van Andel Institute, United States of America

## Abstract

**Background:**

The diversity of functional phenotypes observed within a tumor does not exclusively result from intratumoral genetic heterogeneity but also from the response of cancer cells to the microenvironment. We have previously demonstrated that the morphological and functional phenotypes of melanoma can be dynamically altered upon external stimuli.

**Findings:**

In the present study, transcriptome profiles were generated to explore the molecules governing phenotypes of melanospheres grown in the bFGF(+)EGF(+) serum-free cultures and monolayers maintained in the serum-containing medium. Higher expression levels of MITF-dependent genes that are responsible for differentiation, e.g., *TYR* and *MLANA*, and stemness-related genes, e.g., *ALDH1A1*, were detected in melanospheres. These results were supported by the observation that the melanospheres contained more pigmented cells and cells exerting the self-renewal capacity than the monolayers. In addition, the expression of the anti-apoptotic, MITF-dependent genes e.g., *BCL2A1* was also higher in the melanospheres. The enhanced activity of MITF in melanospheres, as illustrated by the increased expression of 74 MITF-dependent genes, identified MITF as a central transcriptional regulator in melanospheres. Importantly, several genes including MITF-dependent ones were expressed in melanospheres and original tumors at similar levels. The reduced MITF level in monolayers might be partially explained by suppression of the Wnt/β-catenin pathway, and DKK1, a secreted inhibitor of this pathway, was highly up-regulated in monolayers in comparison to melanospheres and original tumors. Furthermore, the silencing of DKK1 in monolayers increased the percentage of cells with self-renewing capacity.

**Conclusions:**

Our study indicates that melanospheres can be used to unravel the molecular pathways that sustain intratumoral phenotypic heterogeneity. Melanospheres directly derived from tumor specimens more accurately mirrored the morphology and gene expression profiles of the original tumors compared to monolayers. Therefore, melanospheres represent a relevant preclinical tool to study new anticancer treatment strategies.

## Introduction

Despite tremendous effort, our understanding of melanoma biology remains insufficient for developing effective therapies for advanced melanoma patients. One of the reasons for this lack of knowledge is the high phenotypic intratumoral heterogeneity and plasticity of melanomas [1]–[4]. Transferring cancer cells from tumor specimens to *in vitro* monolayer cultures markedly alters the biology and response to drugs of tumor cells, and several processes and properties are affected by the growth conditions used to maintain monolayers. As a consequence, the testing of drug efficacy in monolayer cultures has a poor predictive value, with the results differing from those obtained clinically. Therefore, it is crucial to create a preclinical model that more closely mimics the original tumor. In addition to patient-derived tumor xenografts (PDTXs) formed by the transplantation of tumor fragments directly into immunodeficient mice [5]–[7], multicellular spheres maintained *in vitro* in an anchorage-independent manner in a serum-free, growth factors-containing medium are considered better tools than monolayers cultured in the presence of serum [8]–[10]. Nonetheless, it remains a matter of debate whether spheres formed by melanoma cells are a suitable model for the study of melanoma biology [11]–[18]. One of the reasons for the discrepancy in opinions might be the diverse methodologies that are used to obtain and propagate melanospheres but also anchorage-independent cell aggregates. This includes diverse sources of melanoma cells and different compositions of media used for cell culturing. In the present study, melanospheres were derived directly from tumor specimens (PDM, patient-derived melanospheres) and maintained in the bFGF(+)EGF(+) serum-free medium. These melanospheres were found to be enriched with cells with clonogenic potential, reflecting the self-renewal capacity of cancer stem-like cells, and the transition from melanospheres to monolayers was accompanied by a reduction in this property [19]. To identify key pathways and signaling molecules that are important for morphologically and functionally distinguishable phenotypes generated under different growth conditions, we performed a transcriptome analysis and compared the gene expression profiles of patient-derived melanoma cells grown either as three-dimensional melanospheres or as two-dimensional monolayers. In addition, we addressed whether melanospheres better resemble the original tumor than monolayers and demonstrated how easily melanoma cells can change their functional phenotype upon external stimuli.

## Materials and Methods

### Tumor Tissues and Ethics Statement

Nodular melanoma specimens were obtained during surgical procedures and their histopathological characteristics was described previously [19]. This study was approved by the Ethical Commission of the Medical University of Lodz, and written informed consent was obtained from the patients. The melanoma specimens were named DMBC2, DMBC8 and DMBC10 (Department of Molecular Biology of Cancer).

### Cell Culture

Melanoma cells formed anchorage-independent melanospheres in stem cell medium (SCM) consisting of Dulbecco's Modified Eagle's Medium (DMEM)/F12 low-osmolality medium (Lonza, Basel, Switzerland) in the presence of B-27 supplement (Gibco, Paisley, UK), growth factors [10 ng/ml basic fibroblast growth factor (bFGF) and 20 ng/ml epidermal growth factor (EGF); BD Biosciences, San Jose, California, USA], insulin (10 mg/ml), heparin (1 ng/ml), and antibiotics (100 IU/ml penicillin, 100 mg/ml streptomycin, and 2 mg/ml fungizone B). Every few weeks, melanospheres were dissociated for further culturing. To obtain monolayers, the growth factors, insulin and heparin in the culture medium were replaced with 10% fetal bovine serum (FBS).

### Microscopy

The morphology of the melanospheres and monolayers, colonies in agar and cells invading the Matrigel were photographed with a digital Olympus camera (C-5050) attached to an Olympus microscope (CKX41).

### Flow Cytometry

A primary antibody against CD31 (FITC-conjugated) was purchased from eBioscience (San Diego, California, USA), and unconjugated antibodies against Melan-A/MART-1 and gp100 (clone HMB45) were purchased from DAKO (Glostrup, Denmark). Anti-gp100 and anti-Melan-A/MART-1 antibodies were detected with FITC-conjugated goat anti-mouse secondary antibody (BD Pharmingen, San Jose, California, USA). Dead cells were excluded from the analysis by 7-aminoactinomycin D (7-AAD) staining (eBiosciences). Isotype controls were included in each experiment. For intracellular staining, cells were fixed with 4% paraformaldehyde and permeabilized with 0.1% Triton X-100 in PBS for 20 minutes. Typically, 30,000 cells were analyzed. The flow cytometric data were acquired with a FACSCalibur or FACSVersa (BD Biosciences) and analyzed using BD FACSuite software.

### RNA Extraction and Microarrays

Melanospheres were grown in SCM for several weeks until they reached an average size of 300 µm. Cells from the dissociated melanospheres were propagated as monolayers in serum-containing medium for at least 2 weeks prior to harvesting at 90% confluence. Cells were stored in RNAlater solution. Total RNA was extracted using Trizol (Invitrogen Life Technologies, Carlsbad, California, USA) according to the manufacturer's protocol. The RNAs from the melanospheres and their adherent monolayer counterparts were analyzed using the dual-color Human CGH Array G4450A (Agilent Technologies, Santa Clara, California, USA). The data were checked for quality and normalized with no background subtraction. The microarray data related to this paper have been submitted to the Array Express data repository at the European Bioinformatics Institute (http://www.ebi.ac.uk/arrayexpress/) under the accession number E-MTAB-1869. The genes were ordered in a ranked list according to their differential expression (fold change; FC).

### RNA Isolation, cDNA Synthesis and Real-Time PCR

RNA isolation, cDNA synthesis and real-time PCR (qRT-PCR) experiments were performed as described previously [20]. Briefly, RNA was isolated and purified using total RNA isolation kit (A&A Biotechnology, Gdynia, Poland). Subsequently, 1 µg of RNA was transcribed into cDNA by SuperScript II Reverse Transcriptase (Invitrogen Life Technologies, Carlsbad, California, USA). qRT-PCR reactions were performed using KAPA SYBR FAST qPCR kit (Kapa Biosystems, Cape Town, South Africa) and the Rotor-Gene 3000 Real-Time DNA analysis system (Corbett Research, Mortlake, Australia). The annealing temperature for all genes was 56°C. To calculate the relative expression of target genes *versus* a reference gene (*RPS17*), a mathematical model including an efficiency correction for real-time PCR was used. The primers used in the study are listed in Table S1 in File S1.

### Preparation of Cell Lysates and Western Blot Analysis

Cell lysates preparation and immunodetection procedure were described previously [20]. Primary antibodies directed against phosphorylated and total ERK1/2 (Cell Signaling Technology Inc., Danvers, Massachusetts, USA) or β-actin (Sigma-Aldrich, Saint Louis, Missouri, USA) were used, followed by incubation with the secondary HRP-conjugated antibodies (Santa Cruz Biotechnology, Santa Cruz, California, USA).

### Transfection of Melanoma Cells with siRNAs for DKK1 and PRDM1

Melanoma cells from monolayers were seeded in 6-well plates (5×10^5^ cells per well) and cultured in serum-containing medium for 19–24 h, until they reached 30–50% confluence. siRNAs targeting DKK1 or PRDM1 (Santa Cruz Biotechnology, Santa Cruz, California, USA) were transfected using Lipofectamine RNAiMAX (Invitrogen) according to the manufacturer's protocol. To evaluate the off-target effects of the siRNAs, cells were transfected with control siRNA-A (Santa Cruz Biotechnology). The cells were incubated with RNAi duplex-Lipofectamine RNAiMAX complexes for 24 h. The cells were used for the qRT-PCR and clonogenic assays, and the medium was collected for ELISA.

### Soft Agar Colony Formation Assay

Clonogenic assays were performed as described previously [19]. The relative colony-forming capacity was calculated by dividing the number of colonies obtained in the presence of a specific siRNA by the number of colonies generated under control conditions.

### Enzyme-Linked Immunosorbent Assay (ELISA) of DKK1

The ELISA kit Quantikine Human DKK1 Immunoassay (R&D Systems, Minneapolis, Minnesota, USA) was used to determine the concentrations of DKK1 in the culture media. The optical density of each well was determined with a microplate reader (Infinite M200Pro, Tecan, Salzburg, Austria). A standard curve and the concentrations of DKK1 in the samples were obtained by reducing the data using a four-parameter logistic curve fit.

### Invasion Assay

Cell invasion assays were performed using BD BioCoatTM Matrigel Invasion Chambers (BD Biosciences). Melanoma cells (2.5×10^4^) were kept in the Matrigel invasion chambers for 24 h or 48 h. Non-invading cells were removed from the upper surface of the membrane, and the cells on the lower surface were fixed with ethanol, stained with hematoxylin and eosin, microphotographed and counted.

### Microarray Data and Statistical Analysis

The microarray data were log_2_ transformed for a two-class analysis, and *P* values were computed (with *P* values <0.05 used as a cutoff threshold) using Rosetta revolver software. Unsupervised hierarchical clustering accurately separated melanospheres from the adherent monolayers. Fold change (FC) analysis was used to identify genes with expression-level differences between the two classes. The volcanoplot was created using Limma software (version 3.16.4) with a threshold of FC  = 2 and *P* values <0.05. The genes were analyzed with regard to their GO terms by using PANTHER classification system (version 8.1) (http://www.pantherdb.org). The Significance Analysis of Microarray (SAM) was performed using a two-class t-test in the R program (version 3.0.1). The Gene Set Enrichment Analysis (GSEA) of the differentially expressed genes was performed using software downloaded from the Broad Institute (http://www.broadinstitute.org/gsea/index.jsp). Specific biological pathways were defined by the Kyoto Encyclopedia of Genes and Genomes (KEGG) database (www.genome.jp./kegg/). A false discovery rate (FDR) correction not exceeding 0.15 was used as a measure of relevance. In other experiments, the data represent the means ± SD. Student's t-test was used to determine significant differences between the mean values of the tested parameters. The difference was considered significant if *P*<0.05.

## Results

### The Wnt Signaling Pathway- and Melanogenesis-Related Genes Are Differentially Expressed in Melanospheres Grown in Stem Cell Medium and Monolayers Cultured in the Presence of Serum

**Figure 1:**
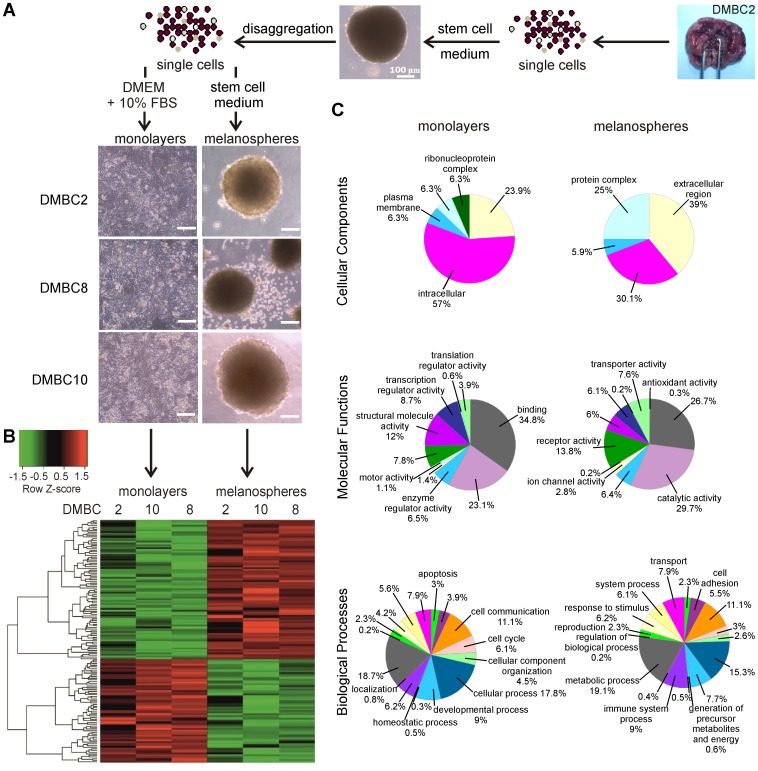
Gene expression profiles of melanoma cells grown as melanospheres and monolayers. **A**. Schematic presentation of the isolation and propagation of 3 patient-derived nodular melanoma cell populations. In SCM, melanoma cells formed highly pigmented, anchorage-independent melanospheres. Cells from dissociated melanospheres transferred to serum-containing medium, grew as adherent monolayers. (Scale bar, 100 µm) **B**. A heatmap of the genes differentially expressed between monolayers and melanospheres was created using log_2_-transformed Z-score values for genes with FC ≥8 as a threshold. **C**. The classification of differentially expressed genes between monolayers and melanospheres based on their functions (Cellular Components, Molecular Functions and Biological Processes) using PANTHER Classification System database. The percentages were calculated as the numbers of gene hits found for melanospheres or monolayers against total number of hits in each category.

Tumor tissues classified as nodular melanomas (NMs), clinical stages III and IV, were obtained to examine differences in the expression profiles of melanoma cells grown either as melanospheres or as monolayers [19]. Highly pigmented densely packed melanospheres with strong cell-cell connections were generated in serum-free SCM containing bFGF and EGF (Fig. 1A). To obtain monolayers for comparative studies, the melanospheres were dissociated, and the melanoma cells were transferred to a serum-containing medium (Fig. 1A).

**Figure 2:**
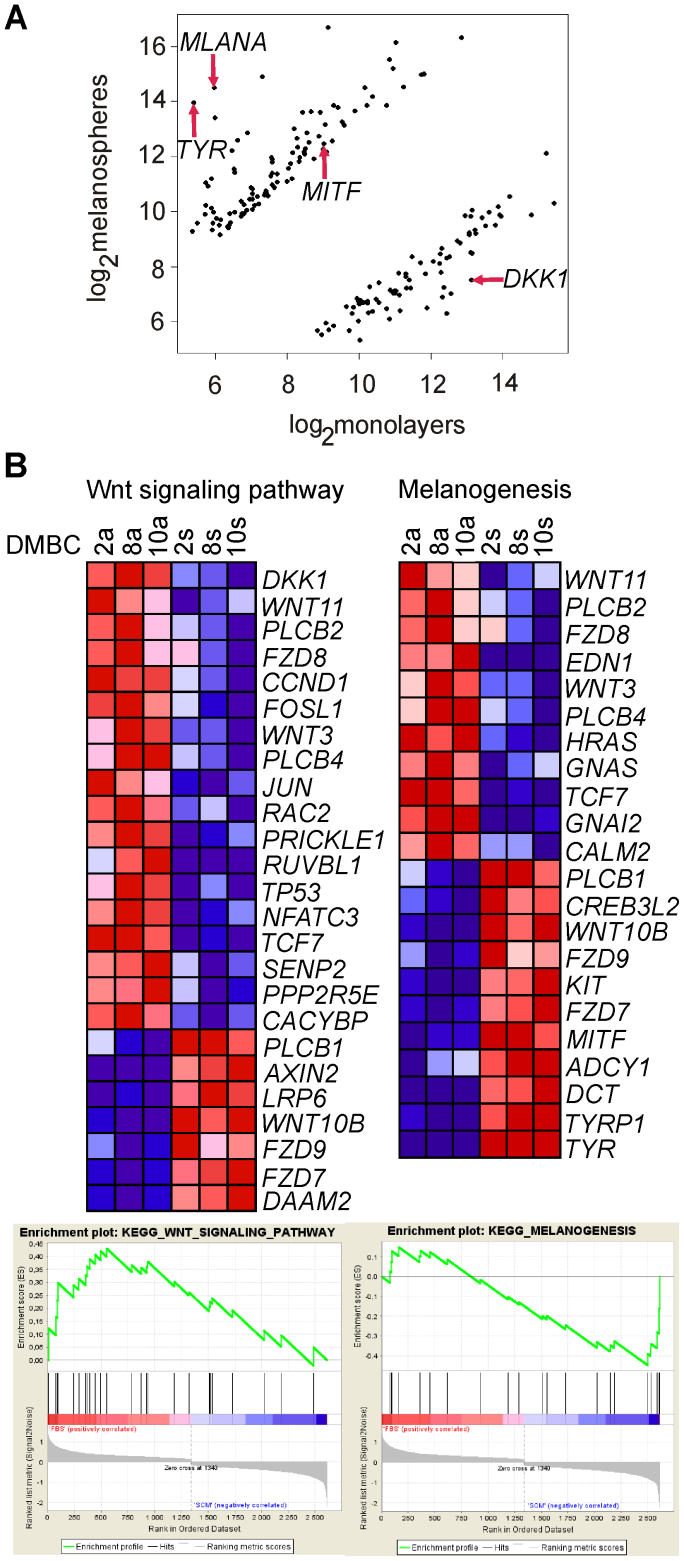
Functional analysis of genes differentially expressed between melanospheres and monolayers. **A**. A Significance Analysis of Microarray (SAM) plot illustrates the signature for differentially expressed genes in melanospheres and monolayers. **B**. Using the KEGG PATHWAY database and the GSEA tool, 25 genes from the microarray analysis were linked to the Wnt pathway (FDR: 0.09, *P* value: 0.007), and 22 genes were linked to melanogenesis (FDR: 0.14, *P* value: 0.03). The heatmaps compare the expression of the indicated genes in monolayers (a; left panels) or melanospheres (s; right panels). Genes in *red* indicate increased expression; genes in *blue* indicate decreased expression.

A microarray analysis identified 1181genes differentially expressed between monolayers and melanospheres, with FC values >2 and *P* values <0.05. A hierarchical clustering of the cells from melanospheres and monolayers (Fig. 1B), indicated that the microenvironment consistently had a strong effect on gene expression across all of the cell lines. The PANTHER Classification System database [21] was used to review the genes with at least twofold differential expression according to their functions (Fig. 1C). With regard to the Cellular Components category, the monolayers showed an enrichment of genes allocated into the intracellular cluster. In contrast, the melanospheres were mostly enriched with gene transcripts coding for ‘protein complexes’ and proteins residing in the ‘extracellular region’, which might partially explain the strong cell-cell interactions in multicellular melanospheres. Interestingly, a cellular component termed ‘ribonucleoprotein complex’ was only allocated to the monolayers. For Molecular Function, a small group of genes in the melanospheres was uniquely annotated to the ‘antioxidant activity’ set. The transcripts giving rise to proteins with ‘transporter activity’, ‘ion channel activity’ and ‘receptor activity’ were more numerous in the melanospheres compared to the monolayer; the latter, in turn, was enriched with transcripts related to ‘translation regulator activity’, ‘structural molecule activity’ and ‘motor activity’. The most prominent gene expression clusters, i.e., ‘binding’ and ‘catalytic activity’, were distributed at a similar level across both phenotypes. When GO terms describing biological processes were analyzed, the samples derived from the melanospheres were enriched with transcripts involved in immune system processes, cell adhesion and generation of precursor metabolites and energy, whereas the samples derived from the monolayers were rich in transcripts responsible for localization, cell cycle and cellular component organization. According to the PANTHER classification, the Wnt (Wingless-type) pathway was the most overrepresented pathway in the melanospheres, with 11 genes being allocated to this pathway corresponding to 4.4% of the total gene hits; 18 genes of the Wnt pathway comprised 4.2% of the gene hits in the monolayers. When a Significance Analysis of Microarray (SAM) was applied to the genes with FC values >8 and *P* values <0.05, 58 genes appeared to be highly up-regulated (FC from 8.2 to 48.3) in the monolayers, and 79 genes were highly up-regulated (FC from 8.0 to 376.7) in the melanospheres (Fig. 2A). The full lists of the affected genes are shown in Tables S2 and S3 in File S1.

The KEGG PATHWAY database and Gene Set Enrichment Analysis (GSEA) were employed to detect biological processes distributed across an entire network of differentially expressed genes. A list of the gene sets that were enriched in the monolayers or melanospheres is shown in Table 1. When the enrichment scores were calculated, 7 gene sets were found to be up-regulated in the monolayers, whereas 2 gene sets were up-regulated in the melanospheres, with nominal *P* values <0.05 and FDR values <0.15. Among these gene sets, 25 genes were linked to the Wnt signaling pathway, which was also one of the “superior pathways” in the PANTHER analysis, and 22 genes were linked to melanogenesis (Fig. 2B). Those 2 gene sets substantially overlapped, as *WNT3, WNT10B, WNT11, FZD7, FZD8, FZD9, TCF7, PLCB1, PLCB2* and *PLCB4* were assigned to both. Notably, the genes that were the most differentially expressed between the melanospheres and monolayers belonged to either of these two pathways. *DKK1*, which encodes a known inhibitory ligand of the Wnt pathway, was the most highly up-regulated gene in the monolayers (FC  = 48.3), whereas tyrosinase (*TYR*; FC  = 376.7) and Melan-A (*MLANA*; FC  = 376.4), which are involved in melanogenesis, were the most highly up-regulated genes in the melanospheres (Tables S2 and S3 in File S1).

**Table 1:** KEGG Pathways.

	NOM	FDR	number of genes expressed in the pathway
	*P* value	*Q* value	
**melanospheres**			
PPAR signaling pathway	0.0208	0.1413	15
Melanogenesis	0.0296	0.1382	22
**monolayers**			
Leukocyte transendothelial migration	0.0025	0.02478	25
Cell cycle	0.0000	0.02154	24
WNT signaling pathway	0.0072	0.09016	25
Neurotrophin signaling pathway	0.0139	0.07820	20
p53 signaling pathway	0.0191	0.06960	16
Pathways in cancer	0.0116	0.14555	60
Cytokine – cytokine receptor interaction	0.0444	0.14126	27

### Differential Expression of Wnt/β-catenin Pathway Components and Target Genes in Melanospheres and Monolayers

**Figure 3:**
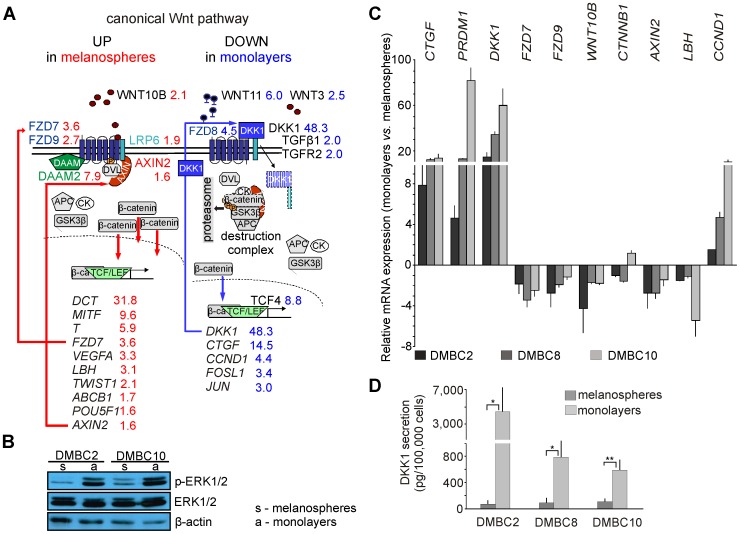
Wnt/β-catenin components and target genes are differentially expressed in melanospheres and monolayers. **A**. The microarray analysis provided several genes related to the canonical Wnt signaling, both its components and targets. FC values in red represent genes up-regulated in melanospheres, and FC values in blue represent genes up-regulated in monolayers. In melanospheres, the interaction of several proteins, including WNT ligands, Frizzled (FZD) receptors, LRP6 co-receptor, Dishevelled (DVL)/AXIN and DAAM2, contribute to the activation of Wnt pathway and the transcription of its target genes, including *MITF*. In monolayers, DKK1 inhibited Wnt signaling by sequestering LRP6. Certain target genes, including *DKK1* and *CTGF*, were up-regulated in monolayers, indicating that other signaling pathways, such as TGFβ/TCF4, might contribute to Wnt pathway activity in this microenvironment. **B**. Activation of MAPK/ERK pathway in melanospheres and monolayers was assessed by monitoring ERK1/2 phosphorylation via western blot analysis. **C**. Expression of selected Wnt pathway genes was validated using qRT-PCR. The expression level of each gene was normalized to the expression of a reference gene, *RPS17*. Data are presented as fold change in monolayers *versus* melanospheres, in which the expression levels of the genes were set as 1. The mean values and SD were calculated from at least 2 experiments performed in triplicates. **D**. ELISA was used to measure DKK1 concentrations in the culture media of melanoma cells grown either as monolayers or as melanospheres. Statistical differences were confirmed using the unpaired t-test; *P*<0.05 (*), *P*<0.01 (**).

To define the molecular background underlying the diverse melanoma cell phenotypes, we focused on the canonical Wnt signaling pathway (Fig. 3A and Table S4 in File S1). LRP6, WNT10B and two Frizzled receptors, FZD7 and FZD9, all of which are recognized as activators of the Wnt/β-catenin pathway, were expressed at higher levels in melanospheres than in monolayers. A recently described enhancer of canonical Wnt signaling, DAAM2, was also up-regulated in melanospheres. Although some positive regulators of this signaling pathway (FZD8, WNT3, RUVBL1 and TCF7) were overexpressed in monolayers, negative regulators, such as SENP2 affecting β-catenin stability, WNT11 and DKK1, a secreted inhibitor of the canonical Wnt pathway, were strongly up-regulated in monolayers. In addition to the genes that were revealed by GSEA, several other Wnt pathway-linked genes were found based on a literature search (Table S4 in File S1). For example, Connective Tissue Growth Factor (CTGF), a mediator of Wnt signaling, was strongly up-regulated in monolayers. Thus, according to the microarray results and the literature-based interpretation of the significance of each component of the canonical Wnt signaling pathway identified in the microarray screening, the microenvironment may substantially influence the composition of Wnt pathway elements, resulting in the expression of different sets of target genes in melanoma cells (Fig. 3A).

In addition, phospho-ERK1/2 was assessed as a readout for activation of the MAPK/ERK pathway. The level of phospho-ERK1/2 was higher in monolayers than in melanospheres (Fig. 3B). This increase in phospho-ERK1/2 was consistent with the enhanced cyclin D1 expression in the monolayers (Fig. 3C).

The expression of selected Wnt pathway genes up-regulated either in melanospheres or in monolayers was validated using qRT-PCR (Fig. 3C). The marked increase in DKK1 expression in monolayers was confirmed by assessing the amount of this protein secreted into the culture medium (Fig. 3D). These results indicate that the Wnt/β-catenin pathway is preferentially active in melanospheres, whereas this pathway is suppressed in monolayers. Moreover, the expression of *PRDM1* (BLIMP1), a transcription factor that might be involved in promoting DKK1 expression, was substantially up-regulated in the monolayers (Fig. 3C, Table S3 in File S1).

### MITF Is a Central Regulator of the Melanosphere Phenotypes

**Figure 4:**
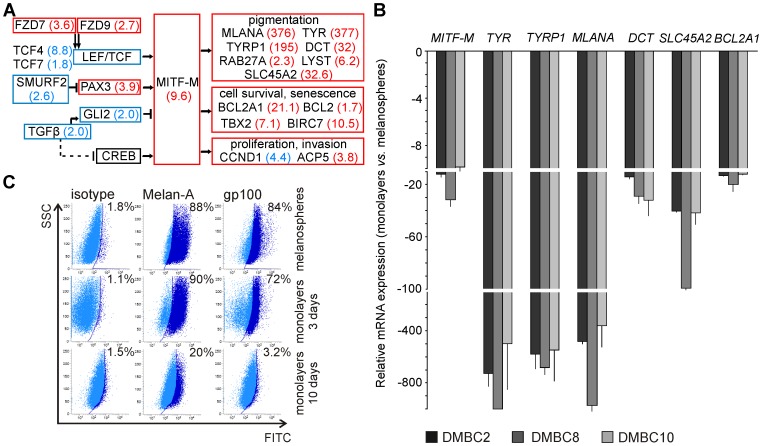
*MITF* and MITF-dependent genes are highly up-regulated in melanospheres. **A**. In a scheme prepared based on the microarray data, only differentially expressed upstream regulators of MITF and downstream target genes are shown. The red boxes and FC values shown in brackets indicate genes that were up-regulated in melanospheres, and the blue boxes, in monolayers. **B**. qRT-PCR validation of *MITF* expression and selected MITF-dependent genes. The expression level of each gene was normalized to the expression of a reference gene, *RPS17*. Data are presented as fold change in monolayers *versus* melanospheres, in which the expression levels of the genes were set as 1. The mean values and SD were calculated from 3 experiments performed in triplicates. A fold change higher than 1000 is shown as 1000. **C**. Representative flow cytometry plots indicate the frequency of Melan-A/MART-1- and gp100-positive cells in melanospheres and monolayers. Flow cytometry-based quantification is shown in Table S6 in File S1.

The microarray data analysis revealed that MITF, a target of the Wnt signaling pathway and a lineage addiction oncogene, was expressed at a higher level in melanospheres compared with monolayers (Fig. 3A). The expression of several genes encoding molecules that regulate the level of MITF differed between melanospheres and monolayers. TGF-β expression was increased in the monolayers (FC  = 2.0), which could result in an increased expression of the MITF inhibitor, GLI2 (FC  = 2.0), and a reduced level of the MITF activator, CREB. The enhanced expression of PAX3 (FC  = 3.9) could contribute to the up-regulation of MITF expression in melanospheres (Fig. 4A). When the expression of MITF-dependent genes was used to assess MITF activity, 74 of these genes were expressed at higher levels in melanospheres than in monolayers (Table S5 in File S1), and 26 of these genes had FC values >5.0. In contrast, only 4 MITF-dependent genes were up-regulated in monolayers, and these 4 had low FC values (between 1.7 and 3.4) (Table S5 in File S1).

Consistently, genes encoding factors involved in the lineage-specific differentiation program were strongly up-regulated in melanospheres, including Melan-A (*MLANA*), enzymes involved in melanin biosynthesis, such as tyrosinase (*TYR*), tyrosinase-related protein 1 (*TYRP-1*) and dopachrome tautomerase (*DCT*, also known as *TYRP-2*), RAB27A, which is essential for normal pigmentation, LYST, a lysosomal trafficking regulator, and SLC45A2, a membrane-associated transporter protein involved in melanin synthesis (Fig. 4A). The microarray data also revealed that melanospheres might be better protected from cell death because the MITF-activated genes that were up-regulated in melanospheres included genes encoding anti-apoptotic proteins, such as BCL2, livin (*BIRC7*) and a lineage-specific anti-apoptotic melanoma oncogene, BCL2A1 (Fig. 4A). The higher expression levels of anti-apoptotic genes as well as *RAB27A* and *GRP143* (Table S5 in File S1) in melanospheres indicate that the response to many anticancer drugs might be artificially enhanced in monolayers. Genes encoding acid phosphatase 5 (ACP5), a transport protein recently recognized as a prognostic biomarker in primary melanomas [22], and T-box 2 (TBX2), a transcription factor that is crucial in embryonic development and in suppressing senescence in melanoma [23], were also up-regulated in melanospheres (Fig. 4A). Notably, the gene encoding GPM6B, a trans-membrane protein whose expression is typically restricted to neuronal tissues, was also expressed at higher levels in melanospheres compared with monolayers (Table S5 in File S1).

Transcriptome profiling indicated that the expression levels of MITF isoform M (MITF-M) and several MITF-dependent genes were strongly up-regulated in the melanospheres, and these results were validated by qRT-PCR. Indeed, *MITF-M* and all of the selected MITF-dependent genes (*TYR, TYRP1, MLANA, DCT, SLC45A2*, and *BCL2A1*) were expressed at significantly (*P<0.05*) higher levels in the melanospheres than monolayers (Fig. 4B). As the expression of some of these genes was strongly inhibited in the monolayers, some by several hundred fold, this result cannot be explained exclusively by the inhibition of gene expression in each cell residing in a melanosphere during the transition to a monolayer. Rather, the obtained results should be considered as average values for the gene expression in melanoma cells comprising melanospheres or monolayers, including all types of subpopulations. Therefore, these data suggest that substantial changes occurred in the composition of subpopulations exerting diverse functional phenotypes. This conclusion is consistent with the reduction in the frequency of Melan-A/MART-1- and gp100-positive cells in the monolayers compared to the melanospheres (Fig. 4C, Table S6 in File S1).

### Melanospheres Resemble the Original Tumor Better Than Monolayers

**Figure 5:**
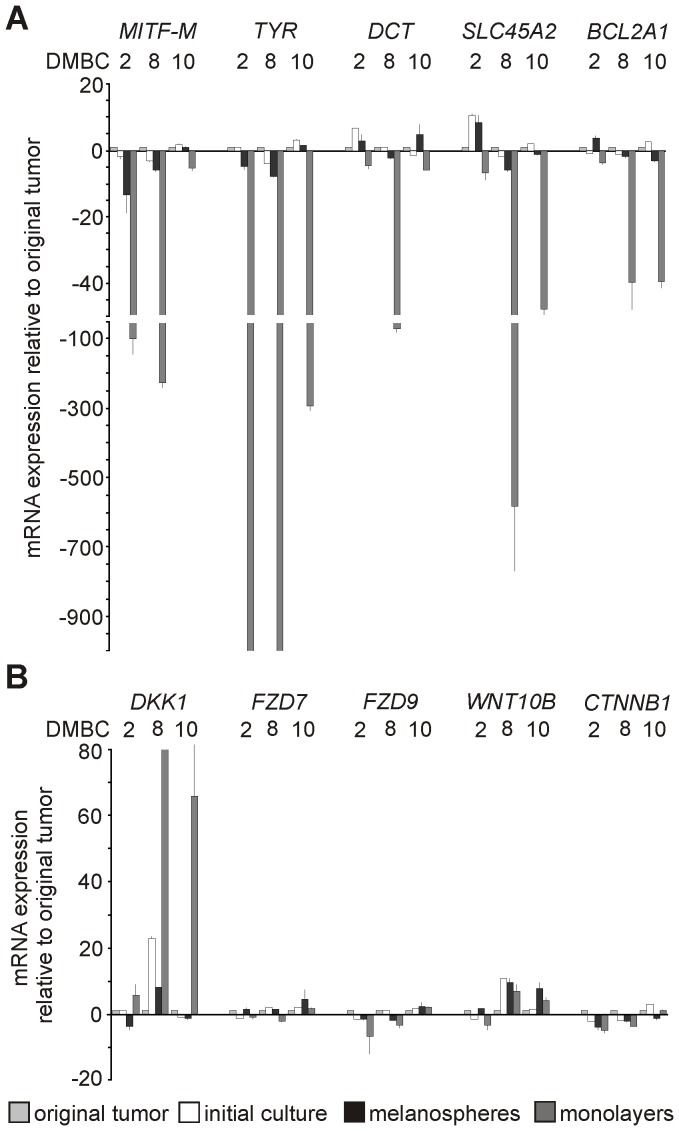
The expression of selected genes in initial short-term cultures (1–3 months), melanospheres and monolayers compared to the expression of these genes in the original tumor. **A**. qRT-PCR validation of the expression of *MITF-M* and selected MITF-dependent genes. **B**. qRT-PCR validation of selected Wnt pathway components or their regulators. The expression level of each gene was normalized to the expression of a reference gene, *RPS17*. Data are presented as fold change in initial culture, melanospheres and monolayers *versus* original tumor, in which the expression levels of the genes were set as 1. The mean values and SD were calculated from at least 2 experiments performed in triplicate.

The expression of the selected genes was also assessed in the original tumors and in initial short-term cultures in SCM (1–3 months). The expression of *MITF-M* and MITF-dependent genes in the original tumors and initial cultures was similar to that obtained in melanospheres but was substantially reduced in monolayers (Fig. 5A). The expression of Wnt pathway components was similar in tumor tissue/initial cultures, melanospheres and monolayers, except for *DKK1* (Fig. 5B). *DKK1* was up-regulated in monolayers compared with the original tumor or melanospheres, suggesting that alterations in the gene expression profiles generated by serum might be, at least in part, due to suppression of the Wnt pathway. These data suggest that melanospheres closely resemble the original tumors, whereas several properties are modified in monolayers.

**Figure 6:**
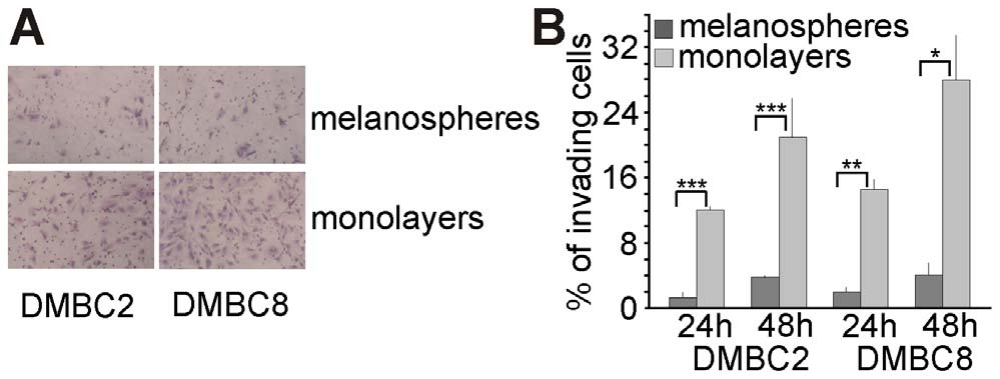
Cells that exert the invasive potential were more frequent in monolayers than in melanospheres. **A**. Monolayers contained more cells capable of invading Matrigel than melanospheres, as shown in representative photomicrographs taken after 24 h. **B**. Invasiveness was measured in Matrigel assays after 24 h and 48 h. Statistically significant differences were confirmed using the unpaired t-test; *P*<0.05 (*), *P*<0.01 (**), *P*<0.001 (***).

Transcriptome profiling supported by qRT-PCR and flow cytometry experiments, revealed that melanospheres contain a subpopulation of highly pigmented differentiated cells that is substantially reduced in monolayers. We have previously demonstrated that melanospheres also contain considerably higher percentages of cancer stem-like cells with self-renewing capacity than monolayers [19]. The current microarray data support this observation as several stemness-related genes, including *ALDH1A1* (FC  = 4.9) [24] and *ABCB1* (FC  = 1.7) [25], were up-regulated in melanospheres. Experiments employing a flow cytometry confirmed that the percentage of ALDH1A1-positive cells was higher in melanospheres than in monolayers (not shown). Moreover, microarray data analyses revealed differential expression of EMT markers, such as the EMT-inducing transcription factor SLUG (*SNAI2*; FC  = 11.1), biglycan (*BGN*; FC  = 8.6) (Table S2 in File S1) and the transcription factor TWIST1 (FC  = 2.1). The melanosphere-associated gene expression signature also included VEGF isoforms, involved in angiogenesis, up-regulated in melanospheres with FC  = 3.3 and FC  = 2.6 for *VEGF-A* and *VEGF-B*, respectively. Monolayers exerted an enhanced invasiveness compared with melanospheres (Fig. 6A/B). The greater invasiveness of the cells in monolayers was consistent with the elevated expression of several genes encoding proteins involved in this process (Table S3 in File S1), including DNA binding protein ID3 with an FC as high as 43.4, non-histone chromatin binding protein HMGA2 (FC  = 19.7), matrix metalloproteinases MMP9 (FC  = 8.6) and MMP2 (FC  = 2.9), the adhesion molecule MCAM (FC  = 4.6) and S100 calcium binding protein A4 (S100A4) (FC  = 17.2). In summary, our study indicates that heterogeneous melanospheres could efficiently execute several functions that are important for tumor development and maintenance, including self-renewal, EMT, angiogenesis and differentiation, whereas monolayers were more uniform.

### Silencing of DKK1 in Monolayers Increased the Percentage of Cells with Self-Renewing Capacity

**Figure 7:**
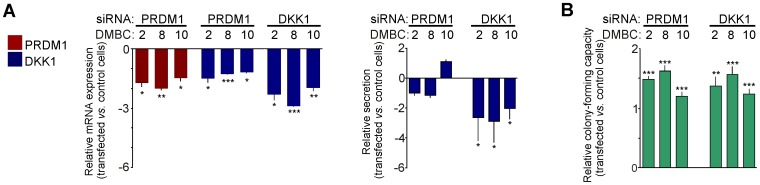
Silencing of DKK1 in monolayers increased the frequency of melanoma cells exerting self-renewing capacity. **A**. Melanoma cells from monolayers were transfected with DKK1 or PRDM1 siRNAs. The transfection efficiency is presented as the relative expression of DKK1 (blue) and PRDM1 (red), as measured by qRT-PCR, and DKK1 secretion, as assessed by ELISA. Data are presented as fold change in cells transfected with targeting siRNA *versus* cells treated with control siRNA, in which the expression levels were set as 1. **B**. Inhibition of DKK1 and PRDM1 enhanced the self-renewing capacity of melanoma cells. The colony-forming capacity of melanoma cells transfected with specific siRNAs is shown relative to that of cells treated with the control siRNA. Bars represent the mean values ± SD.

Next, we attempted to change the phenotype of monolayers by knocking-down the expression of DKK1 or PRDM1 with siRNA. The silencing efficiency, as measured both at the mRNA level and DKK1 secretion, was significant (*P*<0.05) 24 h after transfection, except in the case of DKK1 secretion after silencing with a PRDM1 siRNA (Fig. 7A). To characterize the functional effects of silencing, the self-renewal capacity of melanoma cells was assessed using a clonogenic assay. Cells transfected with DKK1 siRNA or PRDM1 siRNA formed more colonies in soft agar compared with control cells transfected with non-targeting siRNA (Fig. 7B). Thus, silencing either DKK1 or PRDM1 increased the number of cells with self-renewing capacity. However, neither siRNA restored Wnt target gene expression to the levels observed in melanospheres (not shown).

## Discussion

This study demonstrates that patient-derived melanospheres (PDM) represent a simple *in vitro* model to investigate the complexity and plasticity of melanoma. Using a microarray analysis validated by qRT-PCR and functional assays, we provide evidence that melanospheres grown in bFGF(+)EGF(+) stem cell medium not only contain cells with stem cell characteristics but also more closely resemble the original tumor than do monolayers maintained in the presence of serum. In addition, several molecules that have been previously recognized in *in vivo* studies and clinical samples as being crucial for melanoma biology were differentially expressed between melanospheres and monolayers. The changes in the transcriptomes of monolayers *versus* melanospheres were reflected by alterations in the frequencies of melanoma cells that exert diverse functions. The concept of melanoma cell phenotype-switching suggests that those modifications are a consequence of cell interactions with the microenvironment [26].

Functional phenotypes were altered with regard to dominance during the transition from melanospheres to monolayers. The melanospheres contained both primitive cells that had cancer stem-like properties and a large subpopulation of more differentiated cells. This finding is reflected by the enhanced self-renewal capacity of melanospheres [19], their strong pigmentation, high frequency of Melan-A/MART-1- and gp100/PMEL-positive cells and up-regulation of several genes encoding markers of stemness (e.g., *ALDH1A1, ABCB1*) and differentiation (e.g., *TYR*, *DCT*). In this respect, melanospheres differ from the spheres derived from colon cancer [27] and glioblastoma [28] for which low frequencies of cells carrying differentiation markers were found, and from the breast cancer spheres for which the observed clonogenic potential was similar to that of their adherent counterparts [29]. Enhanced self-renewing capacity and tumorigenic potential *in vivo* have already been demonstrated for melanospheres [11], [12], [17]. High percentage of differentiated cells in melanospheres has also been shown [30], however, opposite results have been obtained as well [17], [18]. Fang et al. [11] have shown that patient-derived melanoma spheres that contained tumorigenic cells can be both pigmented or nonpigmented. In the present study, introducing serum to the culture medium reduced the subpopulations of differentiated cells and cells with self-renewal capacity (see also [19]). The alterations in gene expression accompanying these functional changes were considerable reaching a few hundred fold for the genes linked to differentiation.

The reduction of the subpopulations of differentiated cells and cells with self-renewal capacity led to the outgrowth of subpopulations with high proliferation rates [19] and invasiveness (this study). Accordingly, the monolayers exhibited uniquely expressed genes categorized as ‘ribonucleoprotein complex’, which function in DNA replication and RNA splicing, and a more numerous fraction of genes involved in the cell cycle. Several genes encoding proteins involved in proliferation and invasiveness were up-regulated in the monolayers, including *CCND1, GLI2, CTGF, MMP-2, MMP-9, S100A4, TGFB1* and *HMGA2*. It has been shown that CTGF promotes melanoma cell invasion and migration [31], with CTGF inhibition resulting in the opposite effects and reduced MMP-9 expression [32]. S100A4 has been demonstrated as protein regulating tumor growth and angiogenesis in melanoma xenograft model [33] and *S100A4* has shown increased expression in invasive melanoma cell lines [34]. Those more invasive cell lines had also an elevated level of TGFB1 transcript. The activation of TGF-β signaling induced by HMGA2 was shown to occur preferentially at the invasive front of colorectal tumors and in secondary metastatic lesions [35], and in lymph node metastasis of pancreatic adenocarcinoma [36]. Although the role of HMGA2 in melanoma invasiveness is unknown, TGF-β/GLI2 and MITF inversely regulate invasion and pigmentation in melanoma cells [37], [38], which is consistent with the present results. The lower expression of *MITF* and related transcriptional networks correlated with increased invasive potential in a panel of cell lines developed from New Zealand patients with metastatic melanoma [34].

*MITF* was strongly up-regulated in the melanospheres. Moreover, among the 84 genes previously identified as being positively regulated by constitutively active MITF [39], our microarray data indicated that 74 of these genes were expressed at higher levels in melanospheres than monolayers. MITF is considered to be the central transcription factor regulating melanoma phenotypic plasticity [4], and it was reported that *MITF* expression better correlates with the expression of its targets than the *MITF* copy number [40]. We observed significant alterations in *MITF* expression during the serum-induced phenotype switch, confirming the importance of the microenvironment for MITF regulation. Several MITF-dependent genes that were strongly up-regulated in the melanospheres encode proteins responsible for pigmentation. Heavily pigmented metastases were obtained in a melanoma mouse model with the genetically mediated stabilization of β-catenin [41], and we found that several genes involved in differentiation that were up-regulated in that model (e.g., *TYR, TYRP1, DCT, SLC45A, SLC24A, RAB27A*) were also highly expressed in melanospheres. The reduced level of MITF in monolayers might be partially explained by the suppression of the Wnt/β-catenin pathway; indeed, *DKK1*, which encodes a secreted inhibitor of this pathway, was strongly up-regulated in the monolayers. *DKK1* is a target gene of the Wnt pathway, and PRDM1 has recently been proposed to enhance DKK1 levels through an alternative mechanism in glioma cells [42]. In the current study, the silencing of PRDM1 inhibited DKK1 expression but did not significantly reduce DKK1 secretion. DKK1 inhibits melanocyte differentiation and melanin production in epidermis [43], though the role of DKK1 in melanomagenesis is not well characterized. Nonetheless, a reduction in *DKK1* expression was observed in melanoma cell lines compared to melanocytes [44]. Although it has been suggested that DKK1 could be a novel diagnostic/prognostic serum biomarker for a wide range of human cancers [45], serological samples from melanoma patients were not included in that study. More recently, a prospective pilot study [46] revealed that the DKK1 serum levels of melanoma patients were significantly higher than in healthy controls. In light of our findings, a further clinical study is necessary to clarify whether DKK1 could be utilized as a diagnostic serum biomarker for melanoma patients. Furthermore, the silencing of DKK1 in monolayers increased the percentage of clonogenic cells, suggesting a role for Wnt signaling in promoting melanoma cell self-renewal. This finding is in agreement with several studies reporting the function of Wnt signaling in maintaining the pluripotency of embryonic stem cells and cancer development [47]. In melanoma, β-catenin was shown to be an essential survival factor for metastatic cells, whereas it was dispensable for the survival of benign melanocytes and primary, non-invasive cells [48].

In addition to the roles that Wnt/β-catenin signaling plays in melanogenesis and self-renewal, this pathway executes other functions in melanoma. β-catenin suppresses invasion through a cell-type specific mechanism involving MITF, and, interestingly, MITF overexpression in invasive colon cancer cells induces a “melanoma phenotype” [49]. However, another report suggested that β-catenin might be responsible for the induction of melanoma metastasis [50]. In our study, the high monolayer expression of DKK1 was accompanied by increased invasiveness, indicating that the suppression of the Wnt pathway might increase the invasive potential of melanoma cells. Wnt/MAPK crosstalk has also been recognized as a unique interplay in melanoma. Interactions between Wnt signaling and the MAPK/ERK pathway have been described in melanoma and colon and pancreatic cancer, and this crosstalk appears to be cell-type specific [51]–[53]: Wnt signaling inhibits MAPK activity in melanoma yet promotes MAPK activity in the other two tumor types. Our results suggest that this interaction depends on external stimuli in melanoma, as the level of phospho-ERK1/2 was increased in the monolayers maintained in the serum-containing medium. Moreover, it has recently been shown that the BRAF/MAPK pathway suppress MITF expression and activity [54], and the increased phosphorylation of ERK1/2 and the lower MITF expression and activity observed in the monolayers are consistent with those results. The differential expression of Wnt/β-catenin pathway components and target genes in melanospheres (e.g., the gene encoding multi-potent MITF) *versus* monolayers (e.g., the genes encoding CTGF, which is involved in invasiveness, and cyclin D1, which promotes proliferation) suggests that the microenvironment has a strong influence on the functional outcome of the Wnt/β-catenin pathway in melanoma. Further investigation is necessary to delineate the role of Wnt signaling in melanoma cells in diverse *in vivo* microenvironments.

The present results indicate that melanospheres and the original tumors are better equipped to resist cell death than monolayers. The outcome of experiments testing the capacity of a drug to induce cell death would largely depend on the type of *in vitro* culture used, and melanospheres appear to be a better model than monolayers. Most recently, MITF-dependent BCL2A1 was identified as an oncogene restricted to melanoma *versus* normal tissues, and the co-treatment of ^V600E^BRAF melanomas with BRAF inhibitors and obatoclax, an inhibitor of BCL2A1, reduced the resistance to BRAF inhibitors in *BCL2A1*-amplified melanoma cells *in vitro* and *in vivo* [55]. Another study showed that high MITF expression protected melanoma cells against MEK inhibitor cytotoxicity [56]. In our study, *MITF* and *BCL2A1* were expressed in the melanospheres at levels similar to those in the tumor samples, which were higher than in the monolayers. Thus, it is reasonable to assume that melanospheres represent a better *in vitro* model than monolayers to investigate the response of melanoma cells to inhibitors of a lineage-specific MITF-BCL2A1 oncogenic pathway. Our current report and previously published results [57], [58] indicate that melanospheres directly derived from surgical specimens are suitable for studying the interactions between a tumor and its microenvironment, including various compounds with potential anticancer activities. In our most recent study [58], we have re-evaluated compounds from The Natural Products Set II (http://www.dtp.nci.nih.gov) against melanoma cells grown in the conditions suitable for generating melanospheres. In this way, using cultures with the high enough content of melanoma cells with self-renewing capacity, we have selected compounds that are the most potent in affecting cancer stem-like cells exerting this property. Patient-derived melanospheres and melanosphere-generated xenografts have been recently used to investigate targeting of MEK signaling pathway [59]. However, it remains to be determined whether this method could be used to select the most promising modalities as a part of personalized therapeutic decisions. Nonetheless, it will be desirable to investigate the responses of melanoma cells to drugs using patient-derived melanospheres.

## Conclusions

Patient-derived melanospheres, maintained in the bFGF(+)EGF(+) serum-free medium, exert lower expression of DKK1, a Wnt pathway inhibitor, and higher expression of MITF and MITF-dependent genes than do monolayers cultured in the presence of serum. Thus, DKK1 and MITF might be considered as important regulators of microenvironment-driven alterations of melanoma phenotype. As we have shown in the present report and previously published study [19] that the melanospheres contain all types of subpopulations that build the tumor including primitive cells with self-renewing capacity, proliferating cells, fully differentiated pigmented cells and cells with invading capacity, whereas in the monolayers the fractions of self-renewing cells and pigmented cells are substantially diminished following alterations in gene expression pattern, one can speculate that melanospheres are more phenotypically heterogeneous and more accurately resemble the original tumor than monolayers. Thus, patient-derived melanospheres are more suitable model system for the study of melanoma biology and for the screening of various compounds with potential anticancer activities.

## Supporting Information

File S1 **Supporting tables.** **Table S1**, Primer sequences, forward (F) and reverse(R) used in the qRT-PCR study. **Table S2**, Genes up-regulated in melanospheres. Table S3, Genes up-regulated in monolayers. **Table S4**, A: The roles of Wnt pathway-related components linked to this pathway in GSEA. The average fold change (FC) based on the microarray results for 3 patient-derived melanoma samples was determined for the gene expression in cells grown as monolayers in comparison with melanospheres. B: The roles of Wnt pathway-related components linked to this pathway based on literature search. The average fold change (FC) index bases on the microarray results for 3 patient-derived melanoma samples and was determined for the gene expression in monolayers in comparison with melanospheres. **Table S5**, MITF target genes were strongly up-regulated in melanospheres. **Table S6**, Melan-A/MART-1 and gp100 were expressed in melanospheres at higher percentages of cells than in monolayers. The frequencies of Melan A/MART-1- and gp100-positive melanoma cells were assessed by flow cytometry in freshly dissociated melanospheres cultured in SCM and in monolayers maintained in the presence of 10% FBS.
(PDF)
