# Supplementary material for: Gene Expression Profiling Identifies Microphthalmia-Associated Transcription Factor (MITF) and Dickkopf-1 (DKK1) as Regulators of Microenvironment-Driven Alterations in Melanoma Phenotype
Source: PLoS One. 2014 Apr 14;9(4):e95157. doi: 10.1371/journal.pone.0095157 (PMC3986414; doi:10.1371/journal.pone.0095157)
Supplement: File S1 — Supporting tables. Table S1, Primer sequences, forward (F) and reverse(R) used in the qRT-PCR study. Table S2, Genes up-regulated in melanospheres. Table S3, Genes up-regulated in monolayers. Table S4, A: The roles of Wnt pathway-related components linked to this pathway in GSEA. The average fold change (FC) based on the microarray results for 3 patient-derived melanoma samples was determined for the gene expression in cells grown as monolayers in comparison with melanospheres. B: The roles of Wnt pathway-related components linked to this pathway based on literature search. The average fold change (FC) index bases on the microarray results for 3 patient-derived melanoma samples and was determined for the gene expression in monolayers in comparison with melanospheres. Table S5, MITF target genes were strongly up-regulated in melanospheres. Table S6, Melan-A/MART-1 and gp100 were expressed in melanospheres at higher percentages of cells than in monolayers. The frequencies of Melan A/MART-1- and gp100-positive melanoma cells were assessed by flow cytometry in freshly dissociated melanospheres cultured in SCM and in monolayers maintained in the presence of 10% FBS. (PDF) [file pone.0095157.s001.pdf]

Table S1. Primer sequences, forward (F) and reverse(R) used in the qRT-PCR study.

Gene	Sequence	T_M (^oC)	Amplicon (bp)
AXIN2	F: ATGATTCCATGTCCATGACG	56	101
	R: CTTCACACTGCGATGCATTT	56	
BCL2A1	F: GGATAAGGCAAAACGGAGGCTG	62	183
	R: CAGTATTGCTTCAGGAGAGATAGC	59	
CCND1	F: CCCGCACGATTTCATTGAAC	58	134
	R: AGGGCGGATTGGAAATGAAC	58	
CTGF	F: CGACTGGAAGACACGTTTGG	60	249
	R: AGGCTTGGAGATTTTGGGAG	58	
CTNNB1	F: TCTGAGGACAAGCCACAAGATTACA	64	122
	R: TGGGCACCAATATCAAGTCCAA	60	
DCT	F: CTCAGACCAACTTGGCTACAGC	64	113
	R: CAACCAAAGCCACCAGTGTTCC	64	
DKK1	F: CTCGGTTCTCAATTCCAACG	58	172
	R: GCACTCCTCGTCCTCTG	57	
FZD7	F: CAACGGCCTGATGTACTTTAAGG	63	121
	R: CATGTCCACCAGGTAGGTGAGA	64	
FZD9	F: TTCTTCTCCACCGCCTTCAC	60	396
	R: CAGGATGACGATGGTCTTGAG	61	
LBH	F: GGTTCCACCACTATGGAGG	59	183
	R: TCACTGCCCCGACTATCTG	59	
MITF-M	F: GCTGGAAATGCTAGAATACAG	57	379
	R: TTCCAGGCTGATGATGTCATC	59	
MLANA	F: GGACAGCAAAGTGTCTCTTCAAG	63	132
	R: TCAGGTGTCTCGCTGGCTCTTA	64	
PRDM1	F: CAGTTCCTAAGAACGCCAACAGG	62	123
	R: GTGCTGGATTCACATAGCGCATC	62	
RPS17	F: AATCTCCTGATCCAAGGCTG	60	142
	R: CAAGATAGCAGGTTATGTCACG	58	
SLC45A2	F: CTTTGCATCAGCCACCTCATTGG	65	153
	R: TCCAACCTCGACTCCTCTTTCG	64	
TYR	F: CTGGAAGGATTTGCTAGTCCAC	62	106
	R: CCTGTACCTGGGACATTGTTC	61	
TYRP1	F: GAAAAGAGCCACTTTGTCAGGG	62	104
	R: CCATCTGGTCCCAGTATGTCT	61	
WNT10B	F: GAATGCGAATCCACAACAACAG	60	196
	R: TTGCGGTTGTGGGTATCAATGAA	61	

Table S2. Genes up-regulated in melanospheres.

Symbol	Sequence code	FC	Description
TYR	NM_000372	376.68	tyrosinase (oculocutaneous albinism IA)
MLANA	NM_005511	376.38	melan-A
TYRP1	NM_000550	194.94	tyrosinase-related protein 1
PMEL	NM_006928	190.05	premelanosome protein, transcript variant 3
NELL1	NM_006157	62.43	NEL-like 1 (chicken), transcript variant 1
TRIM63	NM_032588	54.27	tripartite motif containing 63
NPTX1	NM_002522	39.42	neuronal pentraxin I
PNLIPRP3	NM_001011709	39.28	pancreatic lipase-related protein 3
IFI27	NM_005532	36.37	interferon, alpha-inducible protein 27, transcript variant 2
HTN1	NM_002159	35.34	histatin 1
VGF	NM_003378	35.13	VGF nerve growth factor inducible
SLC45A2	NM_016180	32.61	solute carrier family 45, member 2, transcript variant 1
DCT	NM_001922	31.76	dopachrome tautomerase (tyrosine-related protein 2), transcript variant 1
FGFBP2	NM_031950	29.45	fibroblast growth factor binding protein 2
SEMA5A	NM_003966	28.21	sema domain, seven thrombospondin repeats (type 1 and type 1-like)
TF	NM_001063	25.89	transferrin (TF)
GPNMB	NM_001005340	25.75	glycoprotein (transmembrane) nmb, transcript variant 1
SOD3	NM_003102	23.80	superoxide dismutase 3, extracellular
D4S234E	NM_014392	22.48	DNA segment on chromosome 4 (unique) 234 expressed sequence, transcript variant 1
CAPN3	NM_000070	21.10	calpain 3, (p94) (CAPN3), transcript variant 1
BCL2A1	NM_004049	21.09	BCL2-related protein A1 (BCL2A1), transcript variant 1
RASD1	NM_016084	20.97	RAS, dexamethasone-induced 1, transcript variant 1
MGP	NM_000900	20.44	matrix Gla protein, transcript variant 2
INSIG1	NM_005542	19.94	insulin induced gene 1, transcript variant
MGP	NM_001190839	19.04	matrix Gla protein (MGP), transcript variant 1
CCDC30	NM_001080850	18.91	coiled-coil domain containing 30
FABP7	NM_001446	18.55	fatty acid binding protein 7, brain
RASEF	NM_152573	18.22	RAS and EF-hand domain containing
CRYAB	NM_001885	17.22	crystallin, alpha B
IRF4	NM_002460	17.07	interferon regulatory factor 4, transcript variant 1
IL33	NM_033439	16.67	interleukin 33, transcript variant 1
CRTAC1	NM_018058	16.25	cartilage acidic protein 1, transcript variant 1
LAMA1	NM_005559	15.14	laminin, alpha 1
CCL18	NM_002988	15.12	chemokine (C-C motif) ligand 18 (pulmonary and activation-regulated)
DNER	NM_139072	14.80	delta/notch-like EGF repeat containing
NUPR1	NM_001042483	14.68	nuclear protein, transcriptional regulator 1, transcript variant 1
PROS1	NM_000313	14.01	protein S (alpha)
ITGB2		13.80	integrin, beta 2 (complement component 3 receptor 3 and 4 subunit)
TNFRSF14	NM_003820	13.65	tumor necrosis factor receptor superfamily, member 14
FAM134B	NM_001034850	13.48	family with sequence similarity 134, member B, transcript variant 1
CKMT1A	NM_001015001	13.43	creatine kinase, mitochondrial 1A, nuclear gene encoding mitochondrial protein

NOX4	NM_016931	13.24	NADPH oxidase 4, transcript variant 1
CCKBR	NM_176875	13.11	cholecystokinin B receptor
CD36	NM_001001547	12.61	CD36 molecule (thrombospondin receptor), transcript variant 2
PI15	NM_015886	12.47	peptidase inhibitor 15
LY96	NM_015364	12.46	lymphocyte antigen 96, transcript variant 1
VCX2	NM_016378	12.28	variable charge, X-linked 2
SLIT3	NM_003062	11.73	slit homolog 3 (Drosophila)
SPARCL1	NM_004684	11.71	SPARC-like 1 (hevin), transcript variant 2
PCSK1	NM_000439	11.60	proprotein convertase subtilisin/kexin type 1, transcript variant 1
PLA2G2F	NM_022819	11.34	phospholipase A2, group IIF
SNAI2	NM_003068	11.10	snail homolog 2 (Drosophila)
SPP1	NM_001040058	11.06	secreted phosphoprotein 1, transcript variant 1
SLC45A2		10.95	solute carrier family 45, member 2
MSMO1	NM_006745	10.81	methylsterol monooxygenase 1, transcript variant 1
PLA1A	NM_015900	10.76	phospholipase A1 member A, transcript variant 1
ASPA	NM_000049	10.58	aspartoacylase (ASPA), transcript variant 1
GPNMB	BC011595,1	10.53	glycoprotein (transmembrane) nmb, mRNA
BIRC7	NM_022161	10.50	baculoviral IAP repeat containing 7, transcript variant 2
TRPM8	NM_014227	10.30	transient receptor potential cation channel, subfamily M, member 8
SLC5A4		10.22	solute carrier family 5 (low affinity glucose transporter), member 4
FKBP11	NM_016594	9.80	FK506 binding protein 11, 19 kDa, transcript variant 1
QPCT	NM_012413	9.64	glutaminyl-peptide cyclotransferase
MITF	NM_198159	9.63	microphthalmia-associated transcription factor, transcript variant 1
P2RX7	NM_002562	9.43	purinergic receptor P2X, ligand-gated ion channel, 7, transcript variant 1
PDK4	NM_002612	9.38	pyruvate dehydrogenase kinase, isozyme 4, nuclear gene encoding mitochondrial protein
IGLL1	NM_020070	9.35	immunoglobulin lambda-like polypeptide 1, transcript variant 1
SERPINF1	NM_002615	9.27	serpin peptidase inhibitor, clade F (pigment epithelium derived factor), member 1
SLC13A3	NM_001011554	9.01	solute carrier family 13, member 3, transcript variant 2
MYB	NM_005375	8.76	v-myb myeloblastosis viral oncogene homolog (avian), transcript variant 2
HRK	NM_003806	8.75	harakiri, BCL2 interacting protein (contains only BH3 domain)
FYB	NM_001465	8.63	FYN binding protein (FYB), transcript variant 1
RAB3A	NM_002866	8.62	RAB3A, member RAS oncogene family
BGN	NM_001711	8.57	biglycan
C1R	NM_001733	8.55	complement component 1, r subcomponent
IL24	NM_001185156	8.40	interleukin 24, transcript variant 3
TKTL1	NM_012253	8.34	transketolase-like 1, transcript variant 1
COLQ	NM_080538	8.27	collagen-like tail subunit of asymmetric acetylcholinesterase, transcript variant 2
DMKN	NM_001035516	8.26	dermokine (DMKN), transcript variant 1

Only genes that showed FC values > 8 with *P* values < 0.05 are included.

Table S3. Genes up-regulated in monolayers.

Symbol	Sequence code	FC	Description
DKK1	NM_012242	48.32	dickkopf homolog 1 (Xenopus laevis)
IGFBP5	NM_000599	46.00	insulin-like growth factor binding protein 5
ID3	NM_002167	43.37	inhibitor of DNA binding 3, dominant negative helix-loop-helix protein
IL7R	NM_002185	41.75	interleukin 7 receptor
CYR61	NM_001554	34.85	cysteine-rich, angiogenic inducer, 61
NUAK1	NM_014840	34.49	NUAK family, SNF1-like kinase, 1
ID1	NM_002165	29.99	inhibitor of DNA binding 1, dominant negative helix-loop-helix protein, transcript variant 1
IGF2BP1	NM_006546	26.88	insulin-like growth factor 2 mRNA binding protein 1 (IGF2BP1), transcript variant 1
LAPTM5	NM_006762	26.70	lysosomal protein transmembrane 5
ANKRD1	NM_014391	26.04	ankyrin repeat domain 1 (cardiac muscle)
STRA6	NM_001199042	25.33	stimulated by retinoic acid gene 6 homolog (mouse), transcript variant 8
MDFI	NM_005586	24.37	MyoD family inhibitor
LIN28B	NM_001004317	22.50	lin-28 homolog B (C. elegans)
HMGA2	NM_003483	19.66	high mobility group AT-hook 2, transcript variant 1
TNFRSF11B	NM_002546	19.43	tumor necrosis factor receptor superfamily, member 11b
S100A4	NM_002961	17.21	S100 calcium binding protein A4 (S100A4), transcript variant 1
KRT18	NM_000224	16.43	keratin 18 (KRT18), transcript variant 1
KRT80	NM_182507	16.38	keratin 80 (KRT80), transcript variant 1
ZFP42	NM_174900	15.97	zinc finger protein 42 homolog (mouse)
VSTM1	NM_198481	15.56	V-set and transmembrane domain containing 1
CLDN4	NM_001305	15.41	claudin 4
S100P	NM_005980	15.17	S100 calcium binding protein P
PMEPA1	NM_020182	15.15	prostate transmembrane protein, androgen induced 1, transcript variant 1
KRT86	NM_002284	14.84	keratin 86
NGFR	NM_002507	14.77	nerve growth factor receptor
BCAT1	NM_005504	14.76	branched chain amino-acid transaminase 1, cytosolic, transcript variant 1
NRP1	NM_003873	14.75	neuropilin 1, transcript variant 1
PLEK2	NM_016445	14.72	pleckstrin 2
CTGF	NM_001901	14.51	connective tissue growth factor
TPM1	NM_000366	14.20	tropomyosin 1 (alpha), transcript variant 5
TPM1	NM_001018005	12.90	tropomyosin 1 (alpha), transcript variant 1
LCTL	NM_207338	12.56	lactase-like
SCHIP1	NM_014575	12.54	schwannomin interacting protein 1, transcript variant 1
EMP1	NM_001423	12.53	epithelial membrane protein 1
THY1	NM_006288	12.53	Thy-1 cell surface antigen
SLITRK6	NM_032229	12.45	SLIT and NTRK-like family, member 6
TPM1	NM_001018004	12.31	tropomyosin 1 (alpha) (TPM1), transcript variant 3
SERPIND1	NM_000185	11.96	serpin peptidase inhibitor, clade D (heparin cofactor), member 1
IL6	NM_000600	11.81	interleukin 6 (interferon, beta 2)
ENC1	NM_003633	11.27	ectodermal-neural cortex 1 (with BTB-like domain)

PPARG	NM_138711	10.89	peroxisome proliferator-activated receptor gamma, transcript variant 3
PRDM1	NM_001198	10.86	PR domain containing 1, with ZNF domain, transcript variant 1
CACNA2D4	NM_172364	10.75	calcium channel, voltage-dependent, alpha 2/delta subunit 4
CD96	NM_198196	10.34	CD96 molecule, transcript variant 1
P2RY6	NM_176798	10.15	pyrimidinergic receptor P2Y, G-protein coupled, 6, transcript variant 2
SERPINB2	NM_002575	10.07	serpin peptidase inhibitor, clade B (ovalbumin), member 2, transcript variant 2
S100A16	NM_080388	9.67	S100 calcium binding protein A16
STRA6	NM_001142620	9.57	stimulated by retinoic acid gene 6 homolog (mouse), transcript variant 5
PAPPA	NM_002581	9.42	pregnancy-associated plasma protein A, pappalysin 1
TCF4	NM_003199	8.84	transcription factor 4, transcript variant 2
LY6K	NM_017527	8.83	lymphocyte antigen 6 complex, locus K, transcript variant 1
ANGPT2	NM_001147	8.70	angiopoietin 2, transcript variant 1
SPOCD1	NM_144569	8.63	SPOC domain containing 1
MMP9	NM_004994	8.60	matrix metallopeptidase 9 (gelatinase B, 92kDa gelatinase, 92kDa type IV collagenase)
SLC16A3	NM_001042422	8.53	solute carrier family 16, member 3 (monocarboxylic acid transporter 4), transcript variant 2
AMOTL2	NM_016201	8.48	angiomotin like 2
IQCJ-SCHIP1	NM_001197113	8.48	IQCJ-SCHIP1 readthrough, transcript variant 1
IL37	NM_014439	8.20	interleukin 37 (IL37). transcript variant 1

Only genes that showed FC > 8.0 with *P* < 0.05 are included.

Table S4

A. The roles of Wnt pathway-related components linked to this pathway in GSEA.
The average fold change (FC) based on the microarray results for 3 patient-derived melanoma samples was determined for the gene expression in cells grown as monolayers in comparison with melanospheres.

Gene	FC	Role in Wnt signaling pathway	References
**up-regulated in monolayers**			
DKK1	48.32	secreted inhibitor of canonical Wnt signaling pathway and its target gene	(1)
WNT11	5.98	inhibitor of β-catenin-dependent signaling<br>activator of non-canonical Wnt signaling pathway	(2,3)
PLCB2	4.92	mediator of Ca^{2+}-dependent Wnt signaling pathway	(4)
FZD8	4.52	activator of canonical Wnt signaling pathway	(5)
CCND1	4.36	target gene of canonical Wnt signaling pathway (cyclin D1)	(6)
RAC2	3.51	mediator of PCP Wnt signaling pathway	(7)
FOSL1	3.41	target gene of canonical Wnt signaling pathway	(8)
JUN	2.98	target gene of canonical Wnt signaling pathway	(8)
PRICKLE1	2.61	inhibitor of canonical Wnt signaling pathway<br>component of PCP Wnt signaling pathway	(10)
WNT3	2.46	activator of both canonical and non-canonical Wnt signaling pathways	(11,12)
RUVBL1	2.31	interacts with β-catenin to enhance β-catenin-dependent gene expression	(13)
PLCB4	2.11	mediator of Ca^{2+}-dependent Wnt signaling pathway	(4)
SENP2	1.99	suppressor of canonical Wnt signaling pathway	(14)
TCF7	1.79	transcription factor regulating gene expression downstream of canonical Wnt signaling pathway	(15)
PPP2R5E	1.78	binds to Axin and inhibits β-catenin-dependent activation of canonical Wnt signaling pathway	(16)
NFATC3	1.67	transcription factor involved in Ca^{2+}-dependent Wnt signaling pathway	(17)
TP53	1.63	suppressor of canonical Wnt signaling pathway	(18)
CACYBP	1.52	suppressor of canonical Wnt signaling pathway	(19)
**up-regulated in melanospheres**			
DAAM2	7.85	enhancer of canonical Wnt signaling pathway	(20)
FZD7	3.56	activator of canonical Wnt signaling pathway and its target gene; contributes to self-renewal of human embryonic stem cells	(21-23)
FZD9	2.74	activator of both canonical and non-canonical Wnt signaling pathways; a marker for human mesenchymal stem cells from placenta	(24-26)
WNT10B	2.09	activator of canonical Wnt signaling pathway; stimulates anchorage-independent growth through non-canonical mechanisms	(27,28)
LRP6	1.90	co-receptor in canonical Wnt signaling pathway activation, necessary for DKK1 inhibitory activity	(29)
PLCB1	1.75	mediator of Ca^{2+}-dependent Wnt signaling pathway	(4)
AXIN2	1.57	negative regulator of β-catenin-dependent signaling and its target gene	(11)

B. The roles of Wnt pathway-related components linked to this pathway based on literature search. The average fold change (FC) index bases on the microarray results for 3 patient-derived melanoma samples and was determined for the gene expression in monolayers in comparison with melanospheres.

Gene	FC	Role in Wnt signaling pathway	References
**up-regulated monolayers**			
CTGF	14.51	target gene of canonical Wnt signaling pathway	(30)
TCF4	8.84	transcription factor regulating gene expression downstream of canonical Wnt signaling pathway	(31)
SHISA9 transcript wariant 1	4.04	prevents Wnt signaling pathway activation through blocking the maturation of Frizzled receptors	(32)
SHISA9 transcript wariant 2	6.74		
RSPO4	2.23	secreted molecule that interferes with DKK1 to activate canonical Wnt signaling pathway	(33)
**up-regulated in melanospheres**			
MITF	9.63	target gene of canonical Wnt signaling pathway	(34)
T (Brachyury)	5.92	target gene of canonical Wnt signaling pathway	(35)
SHISA2	4.34	prevents Wnt signaling pathway activation through blocking the maturation of Frizzled receptors	(32)
VEGFA	3.38	target gene of canonical Wnt signaling pathway	(36)
LBH	3.05	target gene of canonical Wnt signaling pathway	(37)
DAB2	2.16	inhibitor of canonical Wnt signaling pathway through Lrp6-dependent mechanism	(38)
TWIST1	2.07	target gene of canonical Wnt signaling pathway	(39)
ABCB1 (MDR1)	1.66	target gene of canonical Wnt signaling pathway	(40)
POU5F1 (OCT4)	1.60	target gene of canonical Wnt signaling pathway	(41)

References to Table S4

1. Niida, A., Hiroko, T., Kasai, M., Furukawa, Y., Nakamura, Y., Suzuki, Y., 2004. DKK1, a negative regulator of Wnt signaling, is a target of the beta-catenin/TCF pathway. Oncogene 23, 8520-8526.
2. Anton, R., Kestler, H.A., Kuhl, M., 2007. Beta-catenin signaling contributes to stemness and regulates early differentiation in murine embryonic stem cells. FEBS Lett. 581, 5247-5254.
3. Toyama, T., Lee, H.C., Koga, H., Wands, J.R., Kim M., 2002. Noncanonical Wnt11 inhibits hepatocellular carcinoma cell proliferation and migration. Mol. Cancer Res. 8, 254-265.
4. Huelsken J, Behrens J., 2002. The Wnt signalling pathway. J. Cell Sci. 115, 3977-3978.
5. Nam, J.S., Turcotte, T.J., Smith, P.F., Choi, S., Yoon, J.K., 2006. Mouse cristin/R-spondin family proteins are novel ligands for the Frizzled 8 and LRP6 receptors and activate beta-catenin-dependent gene expression. J. Biol. Chem. 281, 13247-13257.
6. Tetsu, O., McCormick, F., 1999. Beta-catenin regulates expression of cyclin D1 in colon carcinoma cells. Nature 398, 422-426.
7. Schlessinger, K., Hall, A., Tolwinski, N., 2009. Wnt signaling pathways meet Rho GTPases. Genes Dev. 23, 265-277.
8. Mann, B., Gelos, M., Siedow, A., Hanski, M.L., Gratchev, A., Ilyas, M., Bodmer, W.F., Moyer, M.P., Riecken, E.O., Buhr, H.J., Hanski, C., 1999. Target genes of beta-catenin-T cell-

factor/lymphoid-enhancer-factor signaling in human colorectal carcinomas. Proc. Natl. Acad. Sci. U. S. A. 96, 1603-1608.
9. Tao, H., Suzuki, M., Kiyonari, H., Abe, T., Sasaoka, T., Ueno, N., 2009. Mouse prickle1, the homolog of a PCP gene, is essential for epiblast apical-basal polarity. Proc. Natl. Acad. Sci. U. S. A. 106,14426-14431.
10. Chan, D.W., Chan, C.Y., Yam, J.W., Ching, Y.P., Ng, I.O., 2006. Prickle-1 negatively regulates Wnt/beta-catenin pathway by promoting Dishevelled ubiquitination/degradation in liver cancer. Gastroenterology 131, 1218-1227.
11. Wu, Y., Ginther, C., Kim, J., Mosher, N., Chung, S., Slamon, D., Vadgama, J.V., 2012. Expression of Wnt3 activates Wnt/beta-catenin pathway and promotes EMT-like phenotype in trastuzumab-resistant HER2-overexpressing breast cancer cells. Mol. Cancer Res. 10, 1597-1606.
12. Kobune, M., Chiba, H., Kato, J., Kato, K., Nakamura, K., Kawano, Y., Takada, K., Takimoto, R., Takayama, T., Hamada, H., Niitsu, Y., 2007. Wnt3/RhoA/ROCK signaling pathway is involved in adhesion-mediated drug resistance of multiple myeloma in an autocrine mechanism. Mol. Cancer Ther. 6, 1774-1784.
13. Bauer, A., Chauvet, S., Huber, O., Usseglio, F., Rothbacher, U., Aragnol, D., Kemler, R., Pradel, J., 2000. Pontin52 and reptin52 function as antagonistic regulators of beta-catenin signalling activity. EMBO J. 19, 6121-6130.
14. Kadoya, T., Kishida, S., Fukui, A., Hinoi, T., Michiue, T., Asashima, M., Kikuchi, A., 2000. Inhibition of Wnt signaling pathway by a novel axin-binding protein. J. Biol. Chem. 275, 37030-37037.
15. Janssens, S., Van Den Broek, O., Davenport, I.R., Akkers, R.C., Liu, F., Veenstra, G.J., Hoppler, S., Vleminckx, K., Destrée, O., 2013. The Wnt signaling mediator tcf1 is required for expression of foxd3 during Xenopus gastrulation. Int. J. Dev. Biol. 57, 49-54.
16. Yamamoto, H., Hinoi, T., Michiue, T., Fukui, A., Usui, H., Janssens, V., Van Hoof, C., Goris, J., Asashima, M., Kikuchi, A., 2001. Inhibition of the Wnt signaling pathway by the PR61 subunit of protein phosphatase 2A. J. Biol. Chem. 20, 26875-26882.
17. de Frutos, S., Spangler, R., Alo, D., Bosc, L.V., 2007. NFATc3 mediates chronic hypoxia-induced pulmonary arterial remodeling with alpha-actin up-regulation. J. Biol. Chem. 282, 15081-15089.
18. Kim, N.H., Kim, H.S., Kim, N.G., Lee, I., Choi, H.S., Li, X.Y., Kang,S.E., Cha, S.,Y., Ryu, J.K., Na, J.M., Park, C., Kim, K., Lee,S., Gumbiner, B.M., Yook, J.I., Weiss, S.J., 2011. p53 and microRNA-34 are suppressors of canonical Wnt signaling. Sci. Signal. 4, ra71.
19. Filipek, A., 2006. S100A6 and CacyBP/SIP - two proteins discovered in ehrlich ascites tumor cells that are potentially involved in the degradation of beta-catenin. Chemotherapy 52, 32-34.
20. Lee, H.K., Deneen, B., 2012. Daam2 is required for dorsal patterning via modulation of canonical Wnt signaling in the developing spinal cord. Dev. Cell 22, 183-196.
21. Nambotin, S.B., Lefrancois, L., Sainsily, X., Berthillon, P., Kim, M., Wands, J.R., Chevallier, M., Jalinot, P., Scoazec, J.Y., Trepo, C., Zoulim, F., Merle, P., 2011. Pharmacological inhibition of Frizzled-7 displays anti-tumor properties in hepatocellular carcinoma. J. Hepatol. 54, 288-299.
22. Melchior, K., Weiss, J., Zaehres, H., Kim, Y.M., Lutzko, C., Roosta, N., Hescheler, J., Muschen, M., 2008. The WNT receptor FZD7 contributes to self-renewal signaling of human embryonic stem cells. Biol. Chem. 389, 897-903.
23. Vincan, E., Flanagan, D.J., Pouliot, N., Brabletz, T., Spaderna, S., 2010. Variable FZD7 expression in colorectal cancers indicates regulation by the tumour microenvironment. Dev. Dyn. 239, 311-317.
24. Karasawa, T., Yokokura, H., Kitajewski, J., Lombroso, P.J., 2002. Frizzled-9 is activated by Wnt-2 and functions in Wnt/beta -catenin signaling. J. Biol. Chem. 277, 37479-37486.
25. Winn, R.A., Marek, L., Han, S.Y., Rodriguez, K., Rodriguez, N., Hammond, M., van Scoyk, M., Acosta, H., Mirus, J., Barry, N., Bren-Mattison, Y., van Raay, T.J., Nemenoff, R.A., Heasley, L.E., 2005. Restoration of Wnt-7a expression reverses non-small cell lung cancer cellular transformation through frizzled-9-mediated growth inhibition and promotion of cell differentiation. J. Biol. Chem. 280, 19625-1934.

26. Battula, V.L., Treml, S., Abele, H., Buhring, H.J., 2008. Prospective isolation and characterization of mesenchymal stem cells from human placenta using a frizzled-9-specific monoclonal antibody. Differentiation 76, 326-336.
27. Modder, U.I., Oursler, M.J., Khosla, S., Monroe, D.G., 2011. Wnt10b activates the Wnt, notch, and NFkappaB pathways in U2OS osteosarcoma cells. J. Cell. Biochem. 112, 1392-1402.
28. Yoshikawa, H., Matsubara, K., Zhou, X., Okamura, S., Kubo, T., Murase, Y., Shihauchi, Y., Esteller, M., Herman, J.G., Wei Wang, X., Harris, C.C., 2007. WNT10B functional dualism: beta-catenin/Tcf-dependent growth promotion or independent suppression with deregulated expression in cancer. Mol. Biol Cell 18, 4292-4303.
29. Bafico, A., Liu, G., Yaniv, A., Gazit, A., Aaronson, S.A., 2001. Novel mechanism of Wnt signalling inhibition mediated by Dickkopf-1 interaction with LRP6/Arrow. Nat. Cell Biol. 3, 683-686.
30. Luo, Q., Kang, Q., Si, W., Jiang, W., Park, J.K., Peng, Y., Li, X., Luu, H.H., Luo, J., Montag, A.G., Haydon, R.C., He, T.C., 2004. Connective tissue growth factor (CTGF) is regulated by Wnt and bone morphogenetic proteins signaling in osteoblast differentiation of mesenchymal stem cells. J. Biol. Chem. 279, 55958-55968.
31. van de Wetering, M., Sancho, E., Verweij, C., de Lau W., Oving, I., Hurlstone, A., van der Horn, K., Batlle, E., Coudreuse, D., Haramis, A.P., Tjon-Pon-Fong, M., Moerer, P., van den Born, M., Soete, G., Pals, S., Eilers, M., Medema, R., Clevers, H., 2002. The beta-catenin/TCF-4 complex imposes a crypt progenitor phenotype on colorectal cancer cells. Cell 111, 241-250.
32. Yamamoto, A., Nagano, T., Takehara, S., Hibi, M., Aizawa, S., 2005. Shisa promotes head formation through the inhibition of receptor protein maturation for the caudalizing factors, Wnt and FGF. Cell 120, 223-235.
33. Kim, K.A., Wagle, M., Tran, K., Zhan, X., Dixon, M.A., Liu, S., Gros, D., Korver, W., Yonkovich, S., Tomasevic, N., Binnerts, M., Abo, A., 2008. R-Spondin family members regulate the Wnt pathway by a common mechanism. Mol. Biol Cell. 19, 2588-2596.
34. Dorsky, R.I., Raible, D.W., Moon, R.T., 2000. Direct regulation of nacre, a zebrafish MITF homolog required for pigment cell formation, by the Wnt pathway. Genes Dev. 14, 158-162.
35. Arnold, S.J., Stappert, J., Bauer, A., Kispert, A., Herrmann, B.G., Kemler, R., 2000. Brachyury is a target gene of the Wnt/beta-catenin signaling pathway. Mech. Dev. 91, 249-258.
36. Zhang, X., Gaspard, J.P., Chung, D.C., 2001. Regulation of vascular endothelial growth factor by the Wnt and K-ras pathways in colonic neoplasia. Cancer Res. 61, 6050-6054.
37. Rieger, M.E., Sims, A.H., Coats, E.R., Clarke, R.B., Briegel, K.J., 2010. The embryonic transcription cofactor LBH is a direct target of the Wnt signaling pathway in epithelial development and in aggressive basal subtype breast cancers. Mol. Cell Biol. 30, 4267-4279.
38. Jiang, Y., He, X., Howe, P.H., 2012. Disabled-2 (Dab2) inhibits Wnt/beta-catenin signalling by binding LRP6 and promoting its internalization through clathrin. EMBO J. 31, 2336-2349.
39. Howe, L.R., Watanabe, O., Leonard, J., Brown, A.M., 2003. Twist is up-regulated in response to Wnt1 and inhibits mouse mammary cell differentiation. Cancer Res. 63, 1906-1913.
40. Correa, S., Binato, R., Du, R.B., Castelo-Branco, M.T., Pizzatti, L., Abdelhay, E., 2012. Wnt/beta-catenin pathway regulates ABCB1 transcription in chronic myeloid leukemia. BMC Cancer 12, 303.
41. Sato, N., Meijer, L., Skaltsounis, L., Greengard, P., Brivanlou, A.H., 2004. Maintenance of pluripotency in human and mouse embryonic stem cells through activation of Wnt signaling by a pharmacological GSK-3-specific inhibitor. Nat. Med. 10, 55-63.

Table S5. MITF target genes were strongly up-regulated in melanospheres.

Symbol	Sequence code	FC	Description	References
**genes up-regulated in melanospheres**				
ACP5	NM_001611	3.8	Acid phosphatase 5, tartrate resistant	(1)
ACSL1	NM_001995	3.4	Acyl-CoA synthetase long-chain 1	(2)
APOE	NM_000041	3.7	Apolipoprotein E	(2)
ASAH1	NM_177924	5.0	N-acylsphingosine amidohydrolase 1	(2)
ATP1A1	NM_000701	2. 4	ATPase, Na+ / K+ transporting, alpha 1 polypeptide	(2)
BCL2	NM_000657	1.7	B-cell CLL / lymphoma 2	(3)
BCL2A1	NM_004049	21.1	BCL2-related protein A1	(2)
BEST1	NM_004183	3.3	Bestrophin 1	(4)
BIRC7	NM_022161	10.5	Baculoviral IAP repeat-containing 7	(5)
CA14	NM_012113	4.2	Carbonic anhydrase XIV	(2)
CAPN3	NM_000070	21.1	Calpain 3	(2)
CDK5R1	NM_003885	2.0	Cyclin-dependent kinase 5, regulatory subunit 1	(2)
CHL1	NM_006614	2.7	Cell adhesion molecule with homology to L1CAM	(2)
CPEB1	NM_030594	2.1	Cytoplasmic polyadenylation element binding protein 1	(2)
CYP27A1	NM_000784	5.4	Cytochrome P450, family 27, subfamily A, polypeptide 1	(2)
DCT	NM_001922	31.8	Dopachrome tautomerase	(6)
DYNC1I1	NM_004411	2.6	Dynein, cytoplasmic 1, intermediate chain 1	(2)
FABP7	NM_001446	18.6	Fatty acid binding protein 7, brain	(2)
FRMD4B	NM_015123	2.7	FERM domain containing 4B	(2)
FXYD3	NM_001136008	5.1	FXYD domain containing ion transport regulator3	(2)
GAPDHS	NM_014364	3.5	Glyceraldehyde-3-phosphate dehydrogenase, spermatogenic	(2)
GCNT2	NM_001491	2.2	Glucosaminyl (N-acetyl) transferase 2, I-branching enzyme	(2)
GNPTAB	NM_024312	2.8	N-acetylglucosamine-1-phosphate transferase a and b	(2)
GPM6B	NM_001001995	5.3	Glycoprotein M6B	(2)
GPNMB	BC011595	10.5	Glycoprotein (transmembrane) nmb	(7)
GPR137B	NM_003272	2.9	G protein-coupled receptor 137B	(2)
GPR143	NM_000273	6.0	G protein-coupled receptor 143 (Oa1)	(8)
GPR56	NM_201525	3.8	G protein-coupled receptor 56	(2)
GREB1	NM_014668	2.6	GREB1 protein	(2)
HPGD	NM_000860	3.8	Hydroxyprostaglandin dehydrogenase 15-(NAD)	(2)
INPP4B	NM_003866	1.6	Inositol polyphosphate-4-phosphatase, type II	(2)
IRF4	NM_002460	17.1	Interferon regulatory factor 4	(2)
ITPKB	NM_002221	4.5	Inositol 1,4,5-trisphosphate 3-kinase B	(2)
KCNN2	NM_021614	2.8	Potassium calcium-activated channel N2	(2)
LGALS3	NM_001177388	2.8	Lectin, galactoside-binding, soluble, 3	(2)
LYST	NM_000081	6.2	Lysosomal trafficking regulator	(2)
LZTS1	NM_021020	3.9	Leucine zipper, putative tumor suppressor 1	(2)
MBP	NM_001025101	6.9	Myelin basic protein	(2)

MDH1	NM_005917	1.8	Malate dehydrogenase 1, NAD (soluble)	(2)
MLANA	NM_005511	376.4	Melan-A	(9)
OSTM1	NM_014028	5.3	Osteopetrosis-associated transmembrane protein 1	(10)
P2RX7	NM_002562	9.4	Purinergic receptor P2X, ligand-gated ion channel	(2)
PIR	NM_003662	3.5	Pirin	(2)
PLA1A	NM_015900	10.8	Phospholipase A1 member A	(2)
QDPR	NM_000320	2.7	Quinoid dihydropteridine reductase	(2)
RAB27A	NM_004580	2.3	RAB27A, member RAS oncogene family	(11)
RRAGD	NM_021244	4.7	Ras-related GTP binding D	(2)
RTP4	NM_022147	4.1	Receptor (chemosensory) transporter protein 4	(2)
SCARB1	NM_005505	2.8	Scavenger receptor class B, member 1	(2)
SCUBE2	NM_020974	1.6	Signal peptide, CUB domain, EGF-like 2	(2)
SGCD	NM_000337	2.5	Sarcoglycan, delta (35kDa dystrophin-associated glycoprotein)	(2)
SIRPA	NM_001040022	4.8	Signal-regulatory protein alpha	(2)
SLC19A2	NM_006996	4.4	Solute carrier family 19 (thiamine transporter), member 2	(2)
SLC1A4	NM_003038	2.2	Solute carrier family 1, member 4	(2)
SLC45A2	ENST00000345083	10.9	Solute carrier family 45, member 2	(13)
SLC7A8	NM_182728	3.3	Solute carrier family 7, member 8	(2)
SORT1	NM_002959	2.1	Sortilin 1	(2)
SPSB1	NM_025106	4.4	splA/ryanodine receptor domain and SOCS box containing 1	(2)
ST3GAL6	NM_006100	3.0	ST3 beta-galactoside alpha-2,3-sialyltransferase 6	(2)
ST8SIA1	NM_003034	6.7	ST8 alpha-N-acetyl-neuraminide alpha-2,8-sialyltransferase 1	(2)
STX7	NM_003569	2.6	Syntaxin	(2)
STXBP1	NM_003165	2.5	Syntaxin binding protein 1	(2)
TBC1D16	NM_019020	2.5	TBC1 domain family, member 16	(2)
TBX2	NM_005994	7.1	T-box 2	(12)
TDRD7	NM_014290	1.9	Tudor domain containing 7	(2)
TIMP2	NM_003255	2.4	TIMP metallopeptidase inhibitor 2	(2)
TMCC2	NM_014858	6.9	Transmembrane and coiled-coil domain family 2	(2)
TMEM51	NM_001136216	2.7	Transmembrane protein 51	(2)
TNFRSF14	NM_003820	13.7	Tumor necrosis factor receptor superfamily 14	(2)
TRIB2	ENST00000405331	6.4	Tribbles homolog 2 (Drosophila)	(2)
TYR	NM_000372	376.7	Tyrosinase	(14)
TYRP1	NM_000550	194.9	Tyrosinase-related protein 1	(15)
UBL3	NM_007106	2.6	Ubiquitin-like 3	(2)
WIPI1	NM_017983	4.9	WD repeat domain, phosphoinositide interacting 1	(2)
**genes up-regulated in monolayers**				
ITGA4	NM_000885	3.4	Integrin 4A	(2)
MTHFS	NM_006441	1.7	5,10-methenyltetrahydrofolate synthetase	(2)
RGS20	NM_170587	2.6	Regulator of G-protein signaling 20	(2)
TMC6	NM_007267	2.9	Transmembrane channel-like 6	(2)

References to Table S5

1. Luchin A, Purdom G, Murphy K, Clark MY, Angel N, et al. (2000) The microphthalmia transcription factor regulates expression of the tartrate-resistant acid phosphatase gene during terminal differentiation of osteoclasts. J Bone Miner Res 15: 451-460.
2. Hoek KS, Schlegel NC, Eichhoff OM, Widmer DS, Praetorius C, et al. (2008) Novel MITF targets identified using a two-step DNA microarray strategy. Pigment Cell Melanoma Res 21: 665-676.
3. McGill GG, Horstmann M, Widlund HR, Du J, Motyckova G, et al. (2002) Bcl2 regulation by the melanocyte master regulator Mitf modulates lineage survival and melanoma cell viability. Cell 109: 707-718.
4. Esumi N, Kachi S, Campochiaro PA, Zack DJ (2007) VMD2 promoter requires two proximal E-box sites for its activity in vivo and is regulated by the MITF-TFE family. J Biol Chem 282: 1838-1850.
5. Dynek JN, Chan SM, Liu J, Zha J, Fairbrother WJ, et al. (2008) Microphthalmia-associated transcription factor is a critical transcriptional regulator of melanoma inhibitor of apoptosis in melanomas. Cancer Res 68: 3124-3132.
6. Yasumoto K, Takeda K, Saito H, Watanabe K, Takahashi K, et al. (2002) Microphthalmia-associated transcription factor interacts with LEF-1, a mediator of Wnt signaling. EMBO J 21: 2703-2714.
7. Loftus SK, Antonellis A, Matera I, Renaud G, Baxter LL, et al. (2009) Gpnmb is a melanoblast-expressed, MITF-dependent gene. Pigment Cell Melanoma Res 22: 99-110.
8. Vetrini F, Auricchio A, Du J, Angeletti B, Fisher DE, et al. (2004) The microphthalmia transcription factor (Mitf) controls expression of the ocular albinism type 1 gene: link between melanin synthesis and melanosome biogenesis. Mol Cell Biol 24: 6550-6559.
9. Du J, Miller AJ, Widlund HR, Horstmann MA, Ramaswamy S, et al. (2003) MLANA/MART1 and SILV/PMEL17/GP100 are transcriptionally regulated by MITF in melanocytes and melanoma. Am J Pathol 163: 333-343.
10. Meadows NA, Sharma SM, Faulkner GJ, Ostrowski MC, Hume DA, et al. (2007) The expression of Clcn7 and Ostm1 in osteoclasts is coregulated by microphthalmia transcription factor. J Biol Chem 282: 1891-1904.
11. Chiaverini C, Beuret L, Flori E, Busca R, Abbe P, et al. (2008) Microphthalmia-associated transcription factor regulates RAB27A gene expression and controls melanosome transport. J Biol Chem 283: 12635-12642.
12. Carreira S, Liu B, Goding CR (2000) The gene encoding the T-box factor Tbx2 is a target for the microphthalmia-associated transcription factor in melanocytes. J Biol Chem 275: 21920-21927.
13. Du J, Fisher DE (2002) Identification of Aim-1 as the underwhite mouse mutant and its transcriptional regulation by MITF. J Biol Chem 277: 402-406.
14. Hou L, Panthier JJ, Arnheiter H (2000) Signaling and transcriptional regulation in the neural crest-derived melanocyte lineage: interactions between KIT and MITF. Development 127: 5379-5389.
15. Fang D, Tsuji Y, Setaluri V (2002) Selective down-regulation of tyrosinase family gene TYRP1 by inhibition of the activity of melanocyte transcription factor, MITF. Nucleic Acids Res 30: 3096-3106.

Table S6. Melan-A/MART-1 and gp100 were expressed in melanospheres at higher percentages of cells than in monolayers.

	melanospheres			monolayers		
	DMBC2	DMBC8	DMBC10	DMBC2	DMBC8	DMBC10
Melan A/MART	81-82	36-80	47-95	3-35	4-20	10-12
gp100 (*PMEL*)	31-88	33-49	66-90	8-22	28-44	7-39

The frequencies of Melan A/MART-1- and gp100-positive melanoma cells were assessed by flow cytometry in freshly dissociated melanospheres cultured in SCM and in monolayers maintained in the presence of 5% FBS.
